# A Multivariate Model for Coastal Water Quality Mapping Using Satellite Remote Sensing Images

**DOI:** 10.3390/s8106321

**Published:** 2008-10-10

**Authors:** Yuan-Fong Su, Jun-Jih Liou, Ju-Chen Hou, Wei-Chun Hung, Shu-Mei Hsu, Yi-Ting Lien, Ming-Daw Su, Ke-Sheng Cheng, Yeng-Fung Wang

**Affiliations:** 1 Department of Bioenvironmental Systems Engineering, National Taiwan University, Taipei, Taiwan.; 2 Hydrotech Research Laboratory, National Taiwan University, Taipei, Taiwan.; 3 Water Resources Agency, Taipei, Taiwan.

**Keywords:** Remote sensing, coastal water quality, environmental monitoring, water quality mapping

## Abstract

This study demonstrates the feasibility of coastal water quality mapping using satellite remote sensing images. Water quality sampling campaigns were conducted over a coastal area in northern Taiwan for measurements of three water quality variables including Secchi disk depth, turbidity, and total suspended solids. SPOT satellite images nearly concurrent with the water quality sampling campaigns were also acquired. A spectral reflectance estimation scheme proposed in this study was applied to SPOT multispectral images for estimation of the sea surface reflectance. Two models, univariate and multivariate, for water quality estimation using the sea surface reflectance derived from SPOT images were established. The multivariate model takes into consideration the wavelength-dependent combined effect of individual seawater constituents on the sea surface reflectance and is superior over the univariate model. Finally, quantitative coastal water quality mapping was accomplished by substituting the pixel-specific spectral reflectance into the multivariate water quality estimation model.

## 1. Introduction

In light of the synoptic spatial coverage and routine availability of satellite images, there have been many implementations of environmental monitoring using remote sensing images in recent years. Among these practices, applications focusing on terrestrial and water environments such as landuse/landcover classification and change detection, landslide site detection, lake and reservoir trophic state monitoring [1-8], ocean harmful algal bloom monitoring [9-12], and coastal water quality monitoring [13-21] are most frequently conducted, although there have been significantly fewer examples of coastal water quality monitoring, as compared to monitoring of impounded water bodies such as lakes and reservoirs. In our opinion, it may be attributed to several factors including difficulty in field data collection, less human activities in the target coastal areas, and the public attitude towards coast utilization and management.

An essential difference in the nature of water quality in coastal area and impounded water bodies is that water quality of impounded water bodies is almost never affected by the downstream condition, whereas coastal water quality is constantly affected by upstream flow discharge and seawater flushing. Upstream inflow may contain high concentration sediments, especially during high-flow periods. Suspended solid material trapped in impounded water bodies can only be removed through overflow from the outfall channel or by other means of surface discharge. For lakes of no flow outlet, suspended solid material carried in by upstream inflow may have long-lasting effect on lake water quality. On the contrary, suspended solid material in coastal water may be mixed or flushed by seawater, and thus the characteristics of creeping impact and low immediate effect make it difficult to sense the emerging consequences which may be severely deleterious and irreversible on coastal ecosystems. For example, Tomascik and Sander [22] and Hoegh-Guldberg *et al.* [23] reported changes to coastal discharge as one of the most serious threats facing coral reef ecosystems worldwide. Thus, understanding the long term effect of sediments carried in the upstream discharge on coastal water quality necessitates a routine monitoring scheme.

Efficient monitoring of the coastal water quality cannot be achieved using only water samples taken in vast coastal area. Not only is the water sampling time and labor consuming, but the required number of samples would also practically hinder such implementation. An integration of remote sensing technique and seawater sampling is therefore more appealing and worth pursuing. Thus, the purpose of this study is to investigate the feasibility of using satellite remote sensing images for coastal water quality monitoring. Specifically, generation of distribution maps of three water quality variables including the total suspended solid (*TSS*), turbidity (*Tb*), and Secchi disk depth (*SDD*) from satellite images is desired.

## 2. Study area and data collection

**Figure 1.**
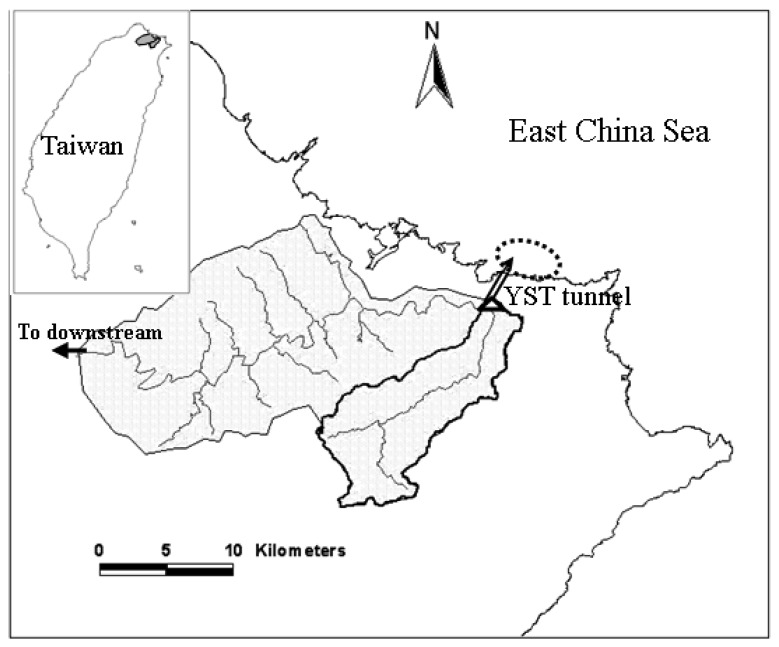
Location map of the study area. Dashed circle indicates the coastal area where water sampling was conducted. Shaded area shows the Keelung River watershed.

**Figure 2.**
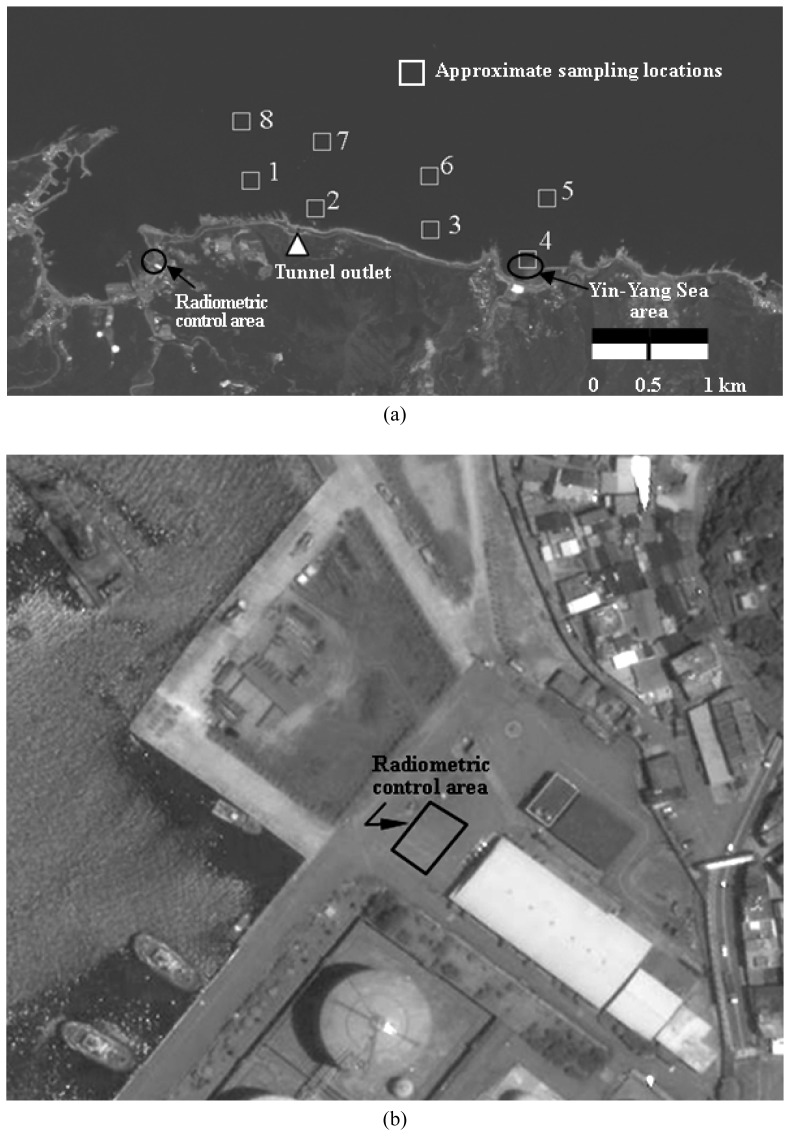
Water sampling locations in the coastal area near the YST tunnel outlet (a) and the radiometric control area (b).

**Figure 3.**
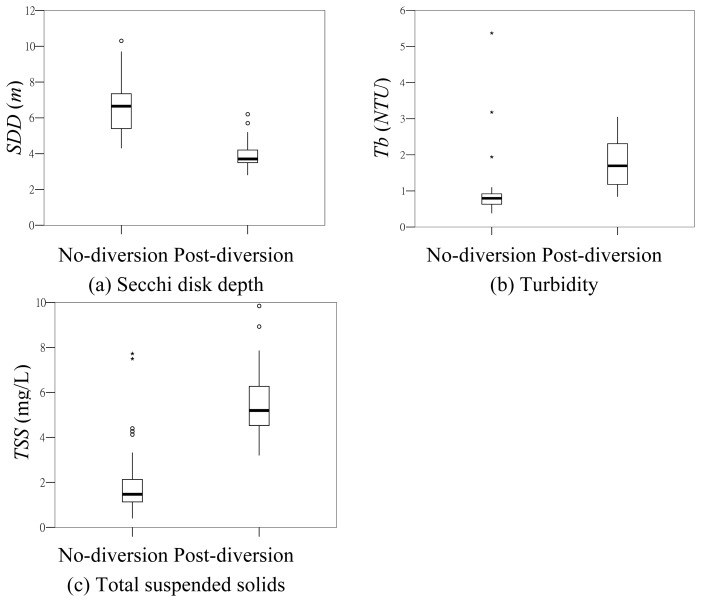
Box plots of water quality data of no-diversion and post-diversion periods.

A coastal area near the outlet of a flood diversion tunnel (the Yuan-Shan-Tzu Diversion Tunnel) in northern Taiwan was chosen for this study (Figure 1). The YST tunnel was constructed to divert flood flow from the upper Keelung River Basin to a discharge outlet at the northern tip of the island. The YST tunnel, completed in 2003 with a diameter of 12 m and 2.48 km in total length, is capable of diverting approximately 81% (1,310 m^3^/s) of the 200-year flood flow (1,620 m^3^/s) at a cross section near the inlet of YST tunnel. Within a five month period (July to November of 2007), a few water sampling campaigns were conducted in a coastal area north of the tunnel outlet. The sampling dates and relevant storm information are shown in Table 1. Water samples were taken within 0 – 20 cm range below the sea surface at eight locations (see Figure 2) during each sampling campaign. Global positioning systems were used to guide the sampling vessel to the desired sampling locations. Considering the west-to-east surface current direction of the season, the sampling area extends from a little northwest of the outlet to about 2 km to the east of the outlet. Sampling point 4 is located within an area known as the Yin-Yang Sea. Geology of the near Yin-Yang Sea area has a large amount of pyrite that does no dissolve easily in water. The Yin-Yang Sea area, just offshore from an old metal mining township, frequently receives runoff containing high iron ion concentration, making the sea surface visually distinct. Secchi disk depth and turbidity were measured *in situ* and water samples were taken to the Environmental Chemistry Lab at the National Taiwan University for analyses of suspended solids and other properties. Table 2 summarizes statistical properties of the three water quality variables. Two sampling campaigns (09/20/2007 and 10/08/2007) took place one day after activation of flood diversion. Comparison of the water quality data of the no-diversion and post-diversion periods is shown in Figure 3. Differences in medians and ranges of *TSS*, *Tb* and *SDD* are apparent. For example, median of *SDD* drops from 6.8 m of the no-diversion period to 3.8 m of the post-diversion period, whereas median of *TSS* increases from 1.6 mg/L of the no-diversion period to 5.2 mg/L of the post-diversion period. Also, excluding the outliers, the ranges of the water quality variables of the no-diversion and post-diversion periods are almost non-overlapping.

**Figure 4.**
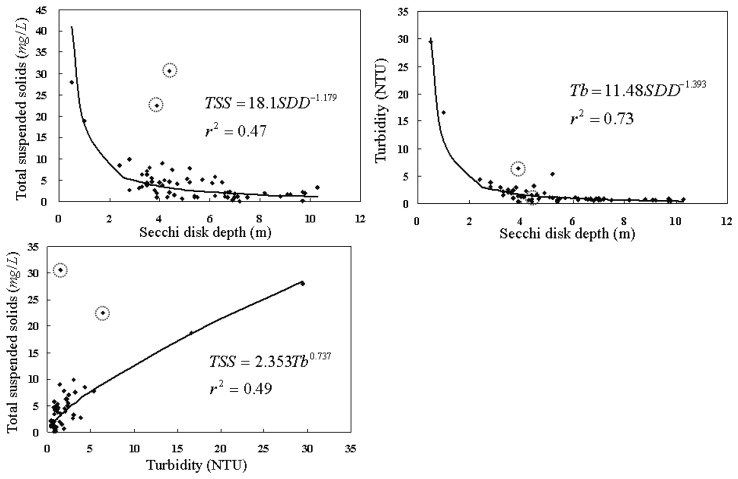
Empirical relationships among different water quality parameters. Points marked by dashed-circles are not included in regression modeling and are excluded from the subsequent analyses.

Before pursuing establishment of water quality estimation models using the water quality data and remote sensing images, we conducted a careful check on measurements of water quality variables. The purpose of such data check is to screen out data which might have been contaminated by inappropriate sampling of water samples or erroneous measurement in the lab. In general, the Secchi disk depth, total suspended solids, and turbidity are inter-related, as demonstrated in Figure 4. The points marked by dashed-circles are significantly inconsistent with such correlation, and thus are excluded in subsequent analyses. Koponen *et al.* [24] also showed similar relationship between the Secchi disk depth and the turbidity.

A few multispectral images from SPOT satellites with acquisition dates close to the dates of sampling campaign were also collected (see Table 1). During and immediately after the Wipha and Krosa typhoon events, the study area were almost completely under cloud cover, and thus no SPOT images were collected. The multispectral SPOT images include images of three spectral bands – green (0.5–0.59μm), red (0.61–0.68μm), and near infrared (0.78–0.89μm), with pixel resolution of 20 m for SPOT-4 or 10 m for SPOT-5.

## 3. Remote sensing image analysis

The amount of solar radiance reaching the satellite sensor can be expressed as [25]:

(1) $$L_{s\lambda }(\theta ,\phi_{d})=(E_{s\lambda }^{\prime }\tau_{1}(\lambda )\cos\sigma \frac{\rho (\sigma ,\phi_{s},\theta ,\phi_{d},\lambda )}{\pi })+F\cdot E_{d\lambda }\frac{\rho (\sigma ,\phi_{s},\theta ,\phi_{d},\lambda )}{\pi }\tau_{2}(\lambda )+L_{u\lambda }(\theta ,\phi_{d})$$

where

*L_sλ_*= solar radiance reaching the sensor
$E_{s\lambda }^{\prime }$ = the exoatmospheric solar irradiance
*E_dλ_* = the downwelled irradiance from the sky dome onto the target
*L_uλ_*= upwelled solar radiance
*θ* = the view angle in the sensor-target direction
*σ* = the sun angle in the sun-target direction
*ϕ_s_* = the azimuth angle in the sun-target direction
*ϕ_d_* = the azimuth angle in the sensor-target direction
*τ*_1_(*λ*) = the atmospheric transmittance along the sun-target path
*τ*_2_(*λ*) = atmospheric transmittance along the target-sensor path.
*λ* = spectral wavelength of solar radiation
*ρ* = spectral reflectance of the target surface
*F* = the obstruction factor.

The obstruction factor *F* in Equation (1) accounts for the proportion of irradiance that may be obstructed by adjacent objects or surface slope of the target. If the target object is on a horizontal surface and free of adjacent object obstruction, the factor *F* equals 1. It is also worthy to note that the sun and view angles are defined with reference to the normal of the target surface. If the target is located on a slope, the sun and view angles will need to be adjusted accordingly. Readers are referred to Schott [25] for detailed calculation of solar radiances arriving at the sensor.

The reflectance *ρ* varies with spectral wavelength and orientation angles. If the target object is assumed to be a diffuse reflector with a constant reflectance *ρ_d_* (*λ*) in all directions, we then have

(2) $$L_{s\lambda }(\theta ,\phi_{d})=(E_{s\lambda }^{\prime }\tau_{1}(\lambda )\cos\sigma +F\cdot E_{d\lambda })\frac{\rho_{d}(\lambda )}{\pi }\tau_{2}(\lambda )+L_{u\lambda }(\theta ,\phi_{d}).$$

On the right-hand side of the above equation, only the spectral reflectance *ρ_d_*(*λ*) represents physical property of the target surface. The upwelled radiance *L_uλ_* does not even get into contact with the target.

Environmental monitoring using remote sensing images often requires derivation of physical properties (reflectance, for example) of the target objects from satellite images. Unfortunately, the upwelled radiance *L_uλ_*, the atmospheric transmittance *τ*_1_ and *τ*_2_, the downwelled irradiance *E_dλ_*, and the exoatmospheric solar irradiance $E_{s\lambda }^{\prime }$ are generally not available for most applications, and we have to resort to other means for estimation of the reflectance.

For most local-scale environmental monitoring applications, *L_uλ_*, *τ*_1_, *τ*_2_, *E_dλ_*, and $E_{s\lambda }^{\prime }$ can be assumed constant (or spatially invariant) within the study area. While on the contrary, the sun angle σ and the obstruction factor *F* are dependent on the surface slope of the target, and the reflectance *ρ_d_*(*λ*) is dependent on surface cover of the earth. Their values may vary from pixel to pixel within a scene. If only pixels on horizontal surface and free of adjacent obstruction are considered (*F* = 1), Equation (1) may be expressed as:

(3) $$\begin{array}{l} L_{s\lambda }(\theta ,\phi_{d})=(E_{s\lambda }^{\prime }\tau_{1}(\lambda )\cos\sigma +E_{d\lambda })\frac{\rho_{d}(\lambda )}{\pi }\tau_{2}(\lambda )+L_{u\lambda }(\theta ,\phi_{d})\\ =k_{1}\rho_{d}(\lambda )+k_{2} \end{array}$$

where $k_{1}=(E_{s\lambda }^{\prime }\tau_{1}(\lambda )\cos\sigma +E_{d\lambda })\frac{\tau_{2}(\lambda )}{\pi }$ and *k*_2_ = *L_uλ_*(*θ*, *ϕ_d_*).

A common practice dealing with the upwelled radiance *L_uλ_*(*θ*, *ϕ_d_*) in satellite remote sensing is the dark object subtraction (DOS) method [5, 26, 27]. The basic concept of the DOS method is to identify very dark features within the scene. The minimum scene radiance is set to be the upwelled radiance based on the assumption that it represents the radiance from a pixel with near zero reflectance. If the minimum scene radiance is subtracted from the radiance of each individual pixel, the processed image is then assumed free of atmospheric scattering effect.

After removing the upwelled radiance *L_uλ_* using the DOS method, the DOS-adjusted radiance $L_{s\lambda }^{\prime }(\theta ,\phi_{d})$ is linearly related to the surface reflectance, i.e.:

(4) $$L_{s\lambda }^{\prime }(\theta ,\phi_{d})=L_{s\lambda }(\theta ,\phi_{d})-L_{u\lambda }(\theta ,\phi_{d})=k_{1}\rho_{d}(\lambda )$$

Based on the linear relationship between $L_{s\lambda }^{\prime }$ and *ρ_d_*(*λ*), we devise a surface reflectance estimation scheme through reflectance calibration in a *radiometric control area* (RCA).

**Figure 5.**
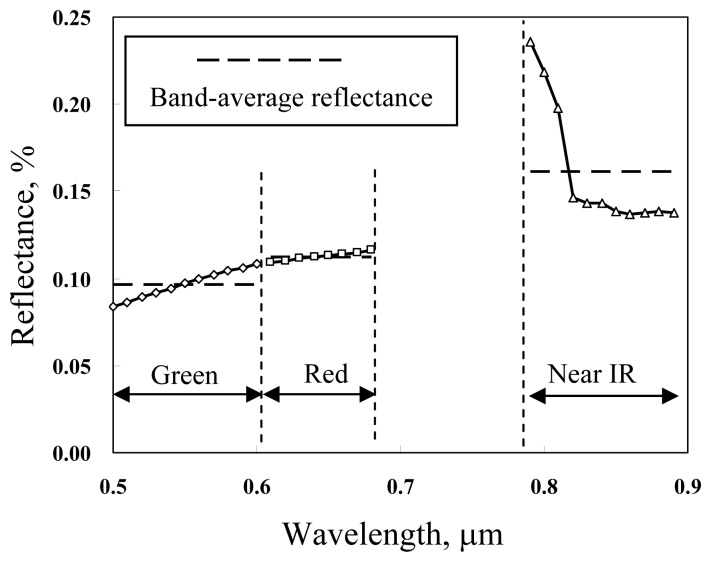
Calibrated RCA-average reflectances and band-average reflectances.

In this study a radiometric control area of approximately 30 m × 60 m was chosen for spectral reflectance calibration. The RCA is a horizontal paved open area with homogeneous and stationary surface reflectance and no adjacent obstruction (see Figure 2). It is located in a restricted and free of public access harbor area. The wavelength-dependent surface reflectance *ρ_d_*(*λ*) of RCA is then calibrated using a variable spectral radiometer (VSR) which is equipped with two spectral-variable filters capable of detecting spectral radiances in various 7 nm-wide windows within the 0.4 – 0.72 μm and 0.65 – 1.1 μm ranges. The VSR was moved around within the radiometric control area taking multispectral images. When taking images within the RCA, a standard reflectance disk which has been pre-calibrated to have *ρ_d_*(*λ*) ≈ 1 over the 0.25 – 1.1 μm wavelength range was also placed within the viewing area. Reflectance of the radiometric control area is then calculated as the ratio of average radiance from RCA to average radiance from the standard reflectance disk, i.e.:

(5) $$\rho_{c}(\lambda )=\frac{{\bar{L}}_{s\lambda ,c}}{{\bar{L}}_{o\lambda }}$$

where *ρ_c_*(*λ*) is the reflectance of RCA, and *L̄_sλ,c_* and *L̄_oλ_* are respectively average radiances received at VSR sensor from the RCA surface and from the standard reflectance disk. For RCA reflectance calibration, the effect of upwelled radiance can be neglected since the VSR is placed near the ground surface. The RCA-average reflectances with respect to various spectral wavelengths are shown in Figure 5. The RCA-average reflectances corresponding to the green, red, and near infrared SPOT spectral bands (hereafter referred to as the RCA band reflectances) are calculated to be 0.097, 0.113, and 0.161%, respectively. The RCA band reflectances are considered constant since the land surface condition within the RCA is relatively homogeneous and stationary.

Assuming the sea surface is horizontal, the DOS-adjusted radiances of pixel *A* on the sea surface and pixel *B* within RCA are respectively expressed by:

(6) $$L_{s\lambda A}^{\prime }(\theta ,\phi_{d})=k_{1}\rho_{dA}(\lambda )$$

and

(7) $$L_{s\lambda B}^{\prime }(\theta ,\phi_{d})=k_{1}\rho_{dB}(\lambda ).$$

Thus, reflectance of the sea surface pixel *A* can be calculated as

(8) $$\rho_{dA}(\lambda )=\frac{L_{s\lambda A}^{\prime }(\theta ,\phi_{d})}{L_{s\lambda B}^{\prime }(\theta ,\phi_{d})}\rho_{dB}(\lambda )$$

In practice, $L_{s\lambda B}^{\prime }(\theta ,\phi_{d})$ and *ρ_dB_*(*λ*) are respectively substituted by the average radiance and reflectance of the RCA, i.e.

(9) $$\rho_{dA}(\lambda )=\frac{\rho_{c}(\lambda )}{{\bar{L}}_{s\lambda ,c}^{\prime }(\theta ,\phi_{d})}L_{s\lambda A}^{\prime }(\theta ,\phi_{d})$$

where ${\bar{L}}_{s\lambda ,c}^{\prime }(\theta ,\phi_{d})$ represents the average value of DOS-adjusted radiances within the RCA and *ρ_c_*(*λ*) is the RCA band reflectance. The reflectance calibration ratio $\frac{\rho_{c}(\lambda )}{{\bar{L}}_{s\lambda ,c}^{\prime }(\theta ,\phi_{d})}$ in the above equation may vary among different SPOT scenes since ${\bar{L}}_{s\lambda ,c}^{\prime }(\theta ,\phi_{d})$ varies due, to scene-to-scene variations in orientation angles and atmospheric transmittance. Table 3 summarizes the reflectance calibration ratios of individual SPOT multispectral images.

## 4. Water quality estimation

In order to map the spatial distribution of the water quality variables using remote sensing images, it is necessary to establish water quality estimation models based on the reflectance of sea surface. A few simple or multiple regression models have been used in the literature [5-8, 13, 14, 21, 28-37]. Most of these models fall into one of the following forms:

(10a) $$\log Y=c_{0}+\sum_{i=1}^{k}c_{i}\log X_{i}(\text{or equivalently},Y=a_{0}\prod_{i=1}^{k}X_{i}^{a_{i}})$$

(10b) $$\log Y=c_{0}+\sum_{i=1}^{k}c_{i}X_{i}$$

(10c) $$Y=c_{0}+\sum_{i=1}^{k}c_{i}X_{i}$$

where *Y* represents a water quality variable and *X_i_* can be reflectance of a specific spectral band, ratio of reflectances of different spectral bands, or other arithmatic calculation of band reflectances.

**Figure 6.**
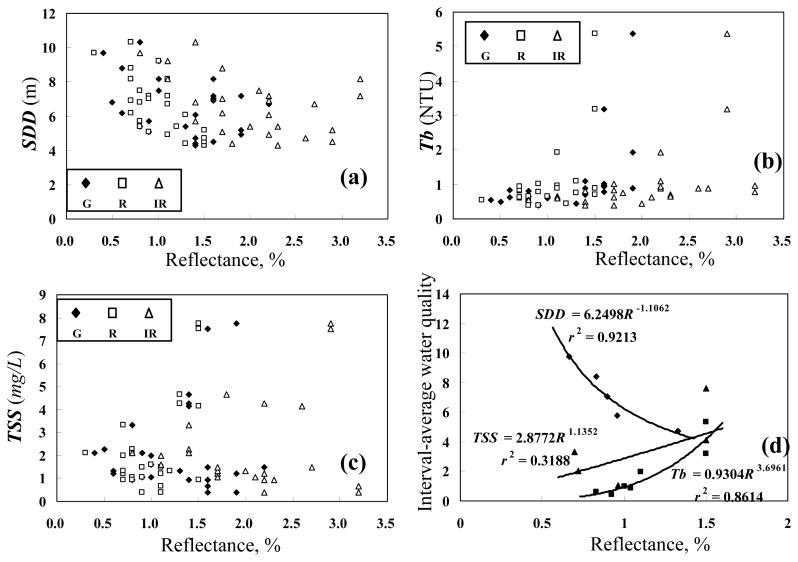
Scatter plot of water quality measurents versus band-dependent sea surface reflectances. (a), (b), (c) Original water quality measurements, (d) interval-average water quality measurements.

In order to choose appropriate models for water quality mapping, we first examined the scatter plots of the water quality measurements versus the band-dependent sea surface reflectances, as shown in Figures 6(a)-(c). Although the data points are widely dispersed, particularly in the lower measurement ranges, measurements of the turbidity and the total suspended solids tend to increase with the sea surface reflectance, whereas the secchi disk depth tends to decrease with increase of the sea surface reflectance. To better illustrate the variation trend of these water quality variables, the water quality measurements were grouped into several incremental intervals. The interval-average water quality and the corresponding average sea surface reflectance were then calculated. Figure 6(d) demonstrates that the interval-average water quality variables are well related to the red band reflectance. Using Equation 10(a), the specific water quality estimation models are as follows:

(11) $$\ln\text{SDD}=1.833-1.106\ln\rho_{R}\left(r^{2}=0.92\right)$$

(12) $$\ln Tb=-0.072+3.696\ln\rho_{R}\left(r^{2}=0.86\right)$$

(13) $$\ln\text{TSS}=1.057+1.135\ln\rho_{R}\left(r^{2}=0.32\right)$$

where *SDD*, *Tb*, and *TSS* are respectively measured in units of m, NTU, and *mg*/*L*, and *ρ_R_* is the red band spectral reflectance in percentage. The signs of regression coefficients of *ρ_R_* in Eqs. (11)–(13) are consistent with the physical phenomena normally observed in the natural environment.

**Figure 7.**
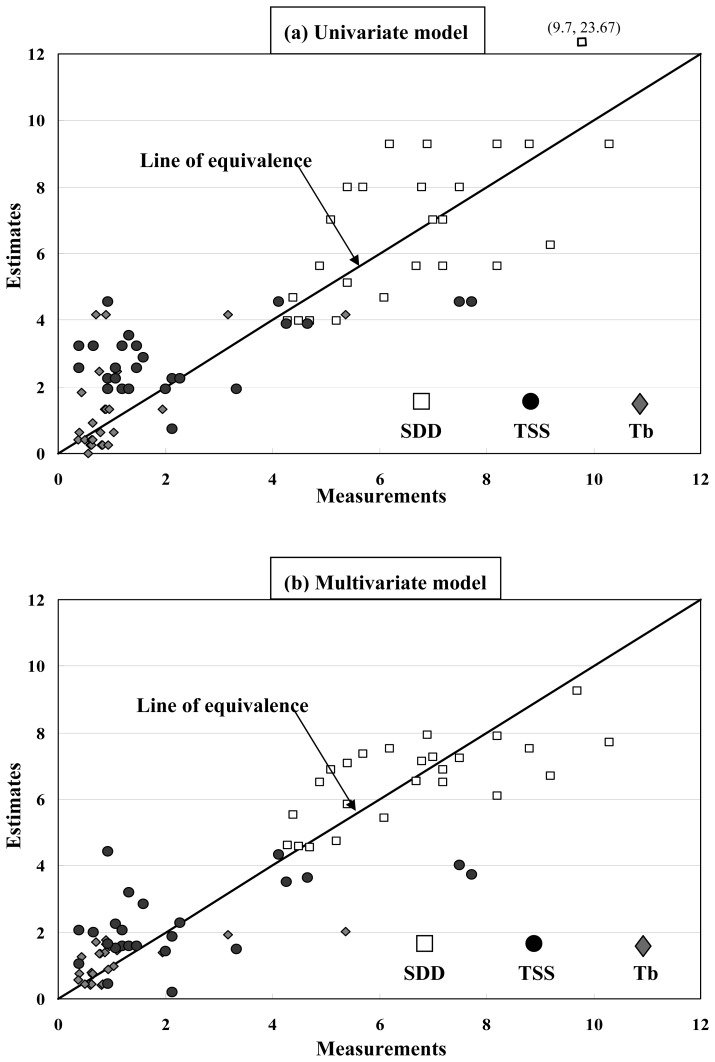
Water quality measurements versus estimates using the univariate and multivariate estimation models. [Note that there is an out-of-bound *SDD* estimate by the univariate model.]

Although the above water quality estimation models were established using the interval-average measurements, these models were adopted for water quality estimation using the pixel-based sea surface reflectance. The applicability of these models was checked by comparing the model estimates against the original measurements. Figure 7(a) demonstrates that the water quality estimates are roughly consistent with the corresponding measurements. Each of the above models utilizes the single band reflectance *ρ_R_* for estimation of a single water quality variable, and is referred to as the *univariate model* in this study.

The water body is a mixture of seawater, suspended solids, dissolved organic matters, zooplankton, etc. The sea surface reflectance of a specific wavelength is affected by the combined effect of these constituents. On the other hand, the effects of individual constituents on the sea surface reflectance vary among different spectral wavelengths. Such *wavelength-dependent combined effect* must be reflected in the water quality estimation model. Thus, we propose the following *multivariate model* for water quality estimation using the multispectral reflectances:

(14) $$\left[\begin{array}{ccc} {\text{SDD}}_{1} & {\text{Tb}}_{1} & {\text{TSS}}_{1}\\ {\text{SDD}}_{2} & Tb_{2} & {\text{TSS}}_{2}\\ \vdots & \vdots & \vdots \\ {\text{SDD}}_{n} & Tb_{n} & {\text{TSS}}_{n} \end{array}\right]=\left[\begin{array}{cccc} 1 & \rho_{G_{1}} & \rho_{R_{1}} & \rho_{IR_{1}}\\ 1 & \rho_{G_{2}} & \rho_{R_{2}} & \rho_{IR_{2}}\\ 1 & \vdots & \vdots & \vdots \\ 1 & \rho_{G_{n}} & \rho_{R_{n}} & \rho_{IR_{n}} \end{array}\right]\cdot \left[\begin{array}{ccc} \beta_{10} & \beta_{20} & \beta_{30}\\ \beta_{11} & \beta_{21} & \beta_{31}\\ \beta_{12} & \beta_{22} & \beta_{32}\\ \beta_{13} & \beta_{23} & \beta_{33} \end{array}\right]+\left[\begin{array}{ccc} {ɛ}_{{\text{SDD}}_{1}} & {ɛ}_{Tb_{1}} & {ɛ}_{{\text{TSS}}_{1}}\\ {ɛ}_{{\text{SDD}}_{2}} & {ɛ}_{Tb_{2}} & {ɛ}_{{\text{TSS}}_{2}}\\ \vdots & \vdots & \vdots \\ {ɛ}_{{\text{SDD}}_{n}} & {ɛ}_{Tb_{n}} & {ɛ}_{{\text{TSS}}_{n}} \end{array}\right]$$

where *β_ij_*'s represent the regression coefficients, *ρ* and *s* are respectively the sea surface reflectance and the random component with respect to the spectral band or the water quality variable shown in their subscripts, and *n* is the number of water quality measurements.

The above multivariate regression model can also be expressed as

(15) Y=XW+E

where **Y**, **X** and **W** are respectively matrices of the water quality measurements, the surface reflectance, and the regression coefficients, and **E** is the error matrix. The least squared estimator of the regression coefficients is [38]:

(16) $$\text{W}={\left(\text{X}\prime \text{X}\right)}^{-1}\text{X}\prime \text{Y}$$

Using a total of 25 samples of water quality measurements and their corresponding multispectral sea surface reflectances, the regression coefficient matrix **W** is estimated to be

(17) $$\text{W}=\left[\begin{array}{ccc} \beta_{10} & \beta_{20} & \beta_{30}\\ \beta_{11} & \beta_{21} & \beta_{31}\\ \beta_{12} & \beta_{22} & \beta_{32}\\ \beta_{13} & \beta_{23} & \beta_{33} \end{array}\right]+\left[\begin{array}{ccc} 10.42 & -0.93 & -0.58\\ 0.54 & 0.32 & -0.97\\ -3.99 & 1.05 & 4.79\\ -0.25 & 0.26 & -0.36 \end{array}\right]$$

Thus, water quality estimates of the multivariate model are obtained by the following equation:

(18) $$\left[\begin{array}{ccc} \text{SDD} & Tb & \text{TSS} \end{array}\right]=\left[\begin{array}{cccc} 1 & \rho_{G} & \rho_{R} & \rho_{IR} \end{array}\right]\cdot \left[\begin{array}{ccc} 10.42 & -0.93 & -0.58\\ 0.54 & 0.32 & -0.97\\ -3.99 & 1.05 & 4.79\\ -0.25 & 0.26 & -0.36 \end{array}\right]$$

Figure 7(b) demonstrates the results of water quality estimation using the multivariate model. Comparing to the estimation results of the univariate model, water quality estimates of the multivariate model show significantly less degree of dispersion around the line of equivalence. Notably, there is an out-of-bound estimate of *SDD* using the univariate model (see Figure 7(a)), whereas the corresponding estimate by the multivariate model is quite accurate. The superior of the multivariate model may be attributed to its capability of modeling the wavelength-dependent combined effect of the seawater constituents. It is also noteworthy that the sign of regression coefficients of the red band reflectance (*ρ_R_*) in both models are consistent, and the red band reflectance remains the dominant factor in the multivariate model, judging from its significantly highest absolute value of the regression coefficient.

## 5. Water quality mapping

**Figure 8.**
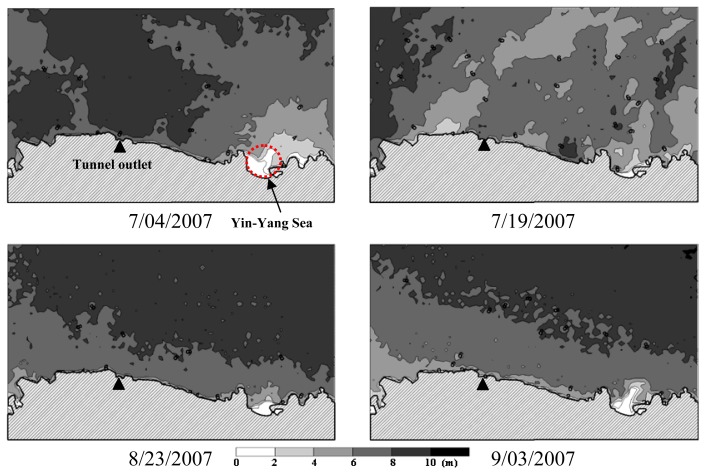
Spatial distribution of secchi disk depth.

**Figure 9.**
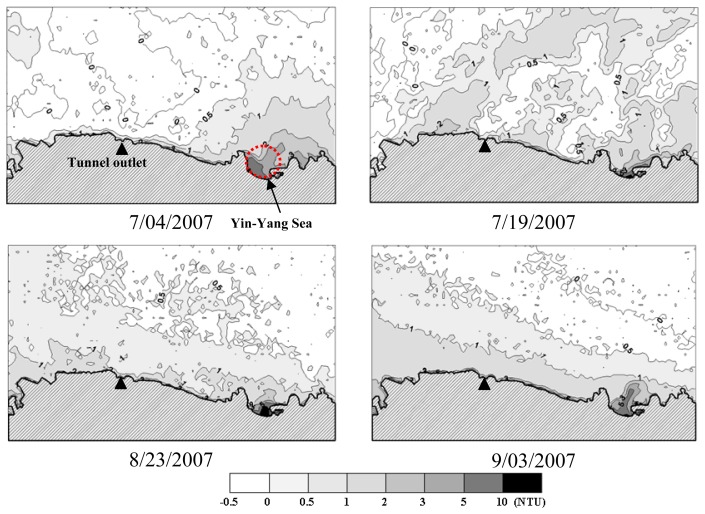
Spatial distribution of turbidity.

**Figure 10.**
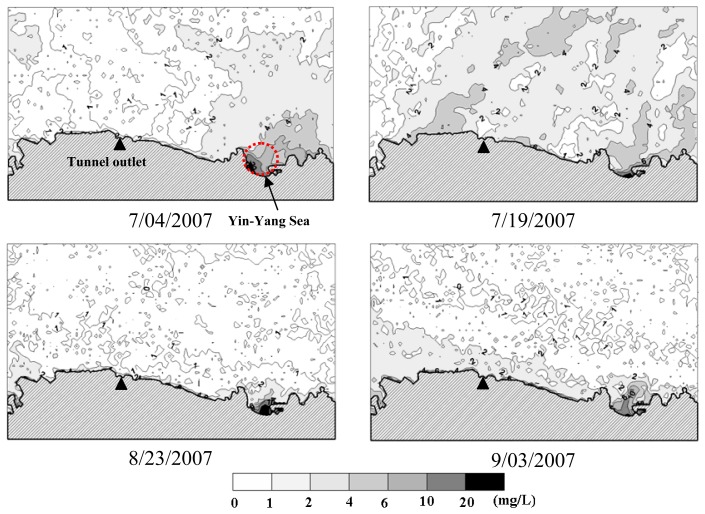
Spatial distribution of total suspended solids.

**Figure 11.**
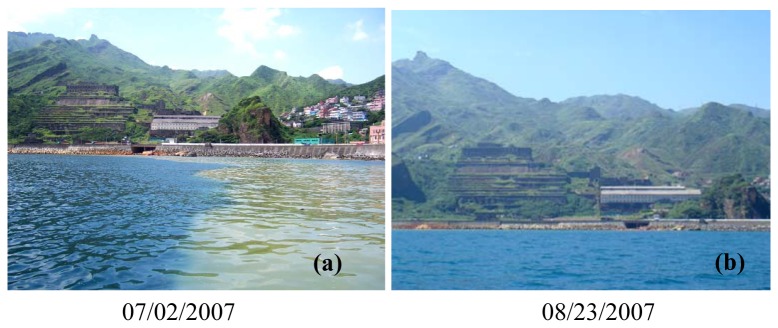
Photos of the Yin-Yang Sea area taken during water sampling campaigns.

Quantitative coastal water quality mapping was accomplished by substituting the pixel-specific spectral reflectance calculated by Eq. (9) into the multivariate model of Equation (18). The resultant water quality distribution maps are shown in Figures 8, 9 and 10. In general, *SDD* increases outward from the near shore area, whereas decreasing *Tb* and *TSS* can be observed. Such spatial variation trends are particularly evident on August 23 and September 3, 2007. Spatial variations of water quality variables on July 4 and 19 appear to be more complicated and the influence of the water quality condition in the Yin-Yang Sea area is more significant. As can be seen in Figure 11 (a), on July 2, 2007 there were higher waves on the sea and the brownish water color (the lower right corner in the figure) suggests higher concentrations of *TSS* and *Tb* in seawater. In contrast, the sea condition was calm on August 23 and no brownish water color can be observed in Figure 11(b). Turbidity distribution mapping also reveals areas with negative *Tb* values. Although small in magnitude, these negative estimates suggest exercising extra caution for estimates of low *Tb* concentration.

Within the study area, the Yin-Yang Sea area has exceptionally higher *TSS* and *Tb* concentrations and lower *SDD* values due to non-dissolved solids routinely received from its upstream area. As for the area offshore from the YST tunnel outlet, no significant effect of the diverted flood flow on coastal water quality has been observed. This may be attributed to very few cases and short duration of flow diversion since the tunnel completion in 2003. However, a monitoring routine using satellite images is recommended for assessing the long term effect of the diverted flood flow on the coastal water quality.

## 6. Conclusions

In this paper we demonstrate the feasibility of coastal water quality mapping using remote sensing images. A few concluding remarks are drawn as follows:

(1) A surface reflectance estimation scheme which involves choosing a radiometric control area was proposed in this study. The scheme is applicable for local-scale environmental monitoring applications.
(2) The three water quality variables (*TSS*, *Tb* and *SDD*) are found to be most related to the red band surface reflectance. High values of the sea surface reflectance generally correspond to high *TSS* and *Tb* concentrations and low *SDD* values.
(3) The water body is a mixture of the seawater and other constituents including the suspended solids, the dissolved organic matters, the zooplankton, etc. The proposed multivariate water quality estimation model takes into consideration the wavelength-dependent combined effect of individual constituents on the sea surface reflectance and yields more accurate water quality estimation results.
(4) Water quality mapping using remote sensing images shows a general pattern of increasing *SDD* and decreasing *Tb* and *TSS* outward from the coast. Under higher wave condition, water quality in the Yin-Yang Sea area may have more significant influence on the spatial distribution of water quality in the nearby area.
(5) Until present, no significant effect of the diverted flow on coastal water quality has been observed due to few cases of flow diversion. However, a routine operation of coastal water quality mapping utilizing satellite images is recommended for assessment of the long term effect of the diverted flow.

## Figures and Tables

**Table 1.** Dates of water sampling and SPOT image acquisition.

Sampling date	SPOT image acquisition date	Relevant storm events	Volume of diverted flow (m^3^)
7/02/2007	7/04/2007 (SPOT-4)	No storm	0
7/18/2007	7/19/2007 (SPOT-4)	No storm	0
8/15/2007	NA^a^	No storm	0
8/23/2007	8/23/2007 (SPOT-5)	Typhoon Sepat (8/16∼8/19)	0
9/07/2007	9/03/2007 (SPOT-5)	No storm	0
9/20/2007	NA^a^	Typhoon Wipha^b^ (9/17∼9/19)	1,051,200
10/08/2007	NA	Typhoon Krosa^b^ (10/4∼10/7)	16,133,400
11/14/2007	NA	No storm	0

a Satellite images were not collected due to high percentage of cloud cover.

b Flow diversion activated.

**Table 2.** Statistical properties of water quality variables.

	Mean	Standard deviation	Maximum	Minimum
Secchi disk depth (*m*)	5.39	2.11	10.30	0.50
Turbidity (*NTU*)	2.19	4.19	29.50	0.38
Total suspended solid (*mg*/*L*)	4.79	5.95	30.61	0

Total number of samples: 62

**Table 3.** Scene reflectance calibration ratios of SPOT multispectral images used in this study.

Image acquisition date	Reflectance calibration ratio $\frac{\rho_{c}(\lambda )}{{\bar{L}}_{s\lambda ,c}^{\prime }(\theta ,\phi_{d})}$		

	Green	Red	Near infrared
7/04/2007	0.00282	0.00313	0.00420
7/19/2007	0.00232	0.00249	0.00410
8/23/2007	0.00331	0.00336	0.00532
9/03/2007	0.00345	0.00342	0.00500
